# Supplementary material for: Interactive Effects of Unhealthy Lifestyle Behaviors on Testicular Function among Healthy Adult Men: A Cross-Sectional Study in Taiwan
Source: Int J Environ Res Public Health. 2021 May 5;18(9):4925. doi: 10.3390/ijerph18094925 (PMC8124479; doi:10.3390/ijerph18094925)
Supplement: Supplementary file 1 [file ijerph-18-04925-s001.zip › ijerph-1172792-supplementary.pdf]

Supplementary Material

Table S1. Factor loading of two dietary patterns identified by principal component analysis.

Food groups	Western-dietary pattern	Prudent-dietary pattern
Eggs	0.451	0.124
Meats	0.542	0.089
Innards organs	0.417	0.165
Rice or flour products cooked in oil	0.458	0.168
Jam or honey	0.410	0.132
Sugary beverages	0.542	-0.114
Deep fried foods	0.680	0.014
Preserved vegetables or processed meat/fish	0.613	0.068
Instant noodles	0.372	-0.072
Dipping sauce	0.609	-0.016
Breads	0.291	0.168
Seafood	0.225	0.374
Legumes or beans	0.239	0.372
Light colored vegetables	-0.071	0.795
Dark or leafy vegetables	-0.09	0.812
Vegetables using oil or salad dressing	0.201	0.566
Fruits	-0.185	0.491
Whole grains	0.036	0.397
Root crops	0.132	0.529
Dairy product	0.24	0.287
Rice or flour product	0.276	0.285
Milk	0.03	0.216
**Total variation explanation**	**27.51%**	
	**14.20%**	**13.31%**

Table S2. Adjusted model of beta (β) coefficients and 95% confidence intervals (CI) of sex hormone biomarkers according to the interaction of lifestyle behaviors.

Lifestyle factors	FSH, IU/L		LH, IU/L		T, ng/mL		E2, pg/mL		Prolactin, ng/mL	
	**β (95% CI)**	***P***	**β (95% CI)**	***P***	**β (95% CI)**	***P***	**β (95% CI)**	***P***	**β (95% CI)**	***P***
**Sleeping type by sleeping time**										
Well										
Enough	Ref		Ref		Ref		Ref		Ref	
Not enough	0.15 (-2.04, 2.34)	NS	0.26 (-0.60, 1.12)	NS	0.13 (-0.26, 0.52)	NS	0.38 (-2.37, 3.13)	NS	-0.12 (-1.35, 1.10)	NS
Insomnia										
Enough	-0.52 (-1.82, 0.77)	NS	-0.10 (-0.62, 0.41)	NS	-0.22 (-0.46, 0.03)	NS	-1.57 (-3.20, 0.06)	NS	0.63 (-0.19, 1.44)	NS
Not enough	0.23 (-1.42, 1.89)	NS	0.07 (-0.58, 0.72)	NS	-0.14 (-0.47, 0.19)	NS	-1.66 (-3.74, 0.41)	NS	-0.33 (-1.68, 1.01)	NS
**Sleeping time by Western dietary pattern**										
Enough										
Low/moderate	Ref		Ref		Ref		Ref		Ref	
High	0.56 (-0.83, 1.95)	NS	0.08 (-0.47, 0.62)	NS	-0.01 (-0.28, 0.25)	NS	-0.79 (-2.54, 0.95)	NS	0.17 (-0.66, 1.01)	NS
Not enough										
Low/moderate	0.62 (-1.02, 2.26)	NS	0.47 (-0.17, 1.11)	NS	0.11 (-0.20, 0.41)	NS	-0.33 (-2.38, 1.72)	NS	-0.40 (-1.62, 0.82)	NS
High	0.92 (-1.21, 3.06)	NS	-0.22 (-1.06, 0.62)	NS	0.06 (-0.36, 0.49)	NS	0.07 (-2.61, 2.75)	NS	-0.45 (-1.80, 0.90)	NS

NS, not significant; FSH, follicle stimulating hormone; LH, luteinizing hormone; T, testosterone; E2, estradiol. Adjusted by age, BMI, FBG, marital status, education level, yearly income, sleeping type, sleeping time, physical activity type, smoking status, and alcohol drinking status.

Table S3. Adjusted model of beta (β) coefficients and 95% confidence intervals (CI) of sperm biomarkers according to interaction of lifestyle behaviors.

Lifestyle factors	SC, M/mL		TSM, %		PRM, %		NSM, %	
	**β (95% CI)**	***P***	**β (95% CI)**	***P***	**β (95% CI)**	***P***	**β (95% CI)**	***P***
**Smoking by sleeping type**								
Not smoker								
Well	Ref		Ref		Ref		Ref	

Insomnia	-0.19 (-3.02, 2.64)	NS	0.25 (-1.38, 1.89)	NS	0.08 (-1.19, 1.36)	NS	-0.25 (-1.72, 1.22)	NS
Smoker								
Well	-1.22 (-4.43, 1.99)	NS	0.89 (-0.97, 2.75)	NS	0.64 (-0.80, 2.09)	NS	-1,14 (-2.80 0.53)	NS
Insomnia	-0.09 (-3.58, 3.40)	NS	1.68 (-0.33, 3.70)	NS	0.99 (-0.58, 2.56)	NS	-1.16 (-2.96, 0.65)	NS
**Smoking by sleeping time**								
Not smoker								
Enough	Ref		Ref		Ref		Ref	
Not enough	-0.77 (-4.53, 2.98)	NS	-0.85 (-2.54, 0.84)	NS	-1.63 (-3.80, 0.54)	NS	-1.57 (-3.51, 0.38)	NS
Smoker								
Enough	-0.43 (-3.18, 2.30)	NS	0.81 (-0.42, 2.04)	NS	0.66 (-0.92, 2.25)	NS	-1.23 (-2.66, 0.19)	NS
Not enough	-2.31 (-6.70, 2.08)	NS	-0.31 (-2.28, 1.66)	NS	1.36 (-1.18, 3.89)	NS	-1.82 (-4.09, 0.46)	NS
**Smoking by Western dietary pattern**								
Not smoker								
Low/moderate	Ref		Ref		Ref		Ref	
High	-3.48 (-6.41, -0.54)	**0.02**	0.02 (-1.30, 1.34)	NS	0.26 (-1.44, 1.96)	NS	-1.49 (-3.01, 0.03)	NS
Smoker								
Low/moderate	-2.38 (-5.91, 1.14)	NS	1.18 (-0.23, 2.59)	NS	1.76 (-0.06, 3.57)	NS	1.04 (-2.67, 0.58)	NS
High	-3.59 (-7.40, 0.22)	NS	0.19 (-1.37, 1.76)	NS	0.48 (-1.54, 2.49)	NS	-2.29 (-4.09, -0.48)	**0.013**
**Drinking by physical activity type**								
Not drinker								
Moderate/intense	Ref		Ref		Ref		Ref	
No/light	0.47 (-2.05, 2.98)	NS	0.31 (-0.82, 1.44)	NS	0.26 (-1.19, 1.71)	NS	-0.49 (-1.79, 0.81)	NS
Drinker								
Moderate/intense	1.36 (-2.94, 5.65)	NS	1.30 (-0.63, 3.22)	NS	-1.51 (-4.04, 1.03)	NS	0.27 (-1.95, 2.49)	NS
No/light	2.83 (-1.55, 7.22)	NS	-0.07 (-2.05, 1.90)	NS	1.51 (-0.97, 3.99)	NS	-2.22 (-4.49, 0.05)	NS
**Drinking by sleeping type**								
Not drinker								
Well	Ref		Ref		Ref		Ref	
Insomnia	-0.08 (-2.59, 2.43)	NS	0.19 (-0.94, 1.32)	NS	0.50 (-0.95, 1.96)	NS	-0.20 (-1.50, 1.10)	NS
Drinker								
Well	0.93 (-3.22, 5.07)	NS	0.47 (-1.40, 2.33)	NS	0.01 (-2.39, 2.40)	NS	-0.82 (-2.97, 1.32)	NS
Insomnia	2.86 (-1.43, 7.15)	NS	0.60 (-1.33, 2.53)	NS	0.18 (-2.30, 2.66)	NS	-0.85 (-3.07, 1.37)	NS
**Drinking by sleeping time**								
Not drinker								
Enough	Ref		Ref		Ref		Ref	
Not enough	-1.52 (-4.73, 1.69)	NS	-0.43 (-1.87, 1.01)	NS	-0.01 (-1.86, 1.84)	NS	-1.61 (-3.27, 0.05)	NS
Drinker								
Enough	1.55 (-1.84, 4.96)	NS	1.00 (-0.53, 2.52)	NS	0.57 (-1.40, 2.54)	NS	-1.21 (-2.97, 0.55)	NS
Not enough	1.65 (-4.59, 7.90)	NS	-2.30 (-5.10, 0.51)	NS	-3.14 (-6.75, 0.47)	NS	-0.42 (-3.65, 2.82)	NS
**Physical activity type by sleeping type**								
Moderate/intense								
Well	Ref		Ref		Ref		Ref	
Insomnia	-0.94 (-4.30, 2.41)	NS	0.21 (-1.30, 1.72)	NS	0.23 (-1.71, 2.17)	NS	0.04 (-1.70, 1.78)	NS
No/light								
Well	-0.31 (-3.29, 2.68)	NS	0.04 (-1.30, 1.38)	NS	-0.48 (-2.21, 1.24)	NS	-0.68 (-2.23, 0.86)	NS
Insomnia	1.01 (-2.23, 4.25)	NS	0.19 (-1.26, 1.65)	NS	0.14 (-1.73, 2.02)	NS	-1.02 (-2.70, 0.65)	NS
**Physical activity type by sleeping time**								
Moderate/intense								
Enough	Ref		Ref		Ref		Ref	
Not enough	-3.03 (-7.60, 1.55)	NS	-1.96 (-4.02, 0.09)	NS	-1.07 (-3.72, 1.57)	NS	-1.49 (-3.86, 0.88)	NS
No/light								
Enough	0.11 (-2.40, 2.62)	NS	-0.28 (-1.41, 0.85)	NS	-0.43 (-1.88, 1.02)	NS	-0.94 (-2.24, 0.37)	NS
Not enough	0.10 (-3.71, 3.91)	NS	-0.57 (-2.28, 1.15)	NS	-0.86 (-3.07, 1.34)	NS	-1.89 (-3.86, 0.08)	NS
**Sleeping type by sleeping time**								
Well								
Enough	Ref		Ref		Ref		Ref	
Not enough	-0.91 (-4.77, 2.95)	NS	-1.23 (-2.97, 0.50)	NS	-0.54 (-2.78, 1.69)	NS	-0.82 (-2.82, 1.18)	NS
Insomnia								
Enough	0.42 (-2.11, 2.94)	NS	0.06 (-1.07, 1.19)	NS	0.51 (-0.95, 1.97)	NS	-0.02 (-1.32, 1.29)	NS
Not enough	-1.21 (-5.44, 3.03)	NS	-0.55 (-2.45, 1.36)	NS	-0.37 (-2.81, 2.08)	NS	1.63 (-3.83, 0.56)	NS

NS, not significant; SC, sperm concentration; TSM, total sperm motility; PRM, progressive motility; NSM, normal sperm morphology. Adjusted by age, BMI, FBG, marital status, education level, yearly income, sleeping type, sleeping time, physical activity type, smoking status, and alcohol drinking status.
